# Whole Genome Sequencing for Tracing Geographical Origin of Imported Cases of Human Brucellosis in Sweden

**DOI:** 10.3390/microorganisms7100398

**Published:** 2019-09-26

**Authors:** Lorena Sacchini, Tara Wahab, Elisabetta Di Giannatale, Katiuscia Zilli, Anna Abass, Giuliano Garofolo, Anna Janowicz

**Affiliations:** 1 National and OIE Reference Laboratory for Brucellosis, Istituto Zooprofilattico Sperimentale dell’Abruzzo e del Molise “G. Caporale”, 64100 Teramo, Italy; lo.sacchini@izs.it (L.S.); e.digiannatale@izs.it (E.D.G.); k.zilli@izs.it (K.Z.); a.abass@izs.it (A.A.); a.janowicz@izs.it (A.J.); 2 Department of Microbiology, Public Health Agency of Sweden, 171 82 Solna, Sweden; tara.wahab@folkhalsomyndigheten.se

**Keywords:** brucellosis, *Brucella melitensis*, WGS, MLST, MLVA, cgMLST

## Abstract

Human infections with *Brucella melitensis* are occasionally reported in Sweden, despite the fact that the national flocks of sheep and goats are officially free from brucellosis. The aim of our study was to analyze 103 isolates of *B. melitensis* collected from patients in Sweden between 1994 and 2016 and determine their putative geographic origin using whole genome sequencing (WGS)-based tools. The majority of the strains were assigned to East Mediterranean and African lineages. Both *in silico* Multiple Loci VNTR (Variable Number of Tandem Repeats) Analysis (MLVA) and core genome Multilocus Sequence Typing (cgMLST) analyses identified countries of the Middle East as the most probable source of origin of the majority of the strains. Isolates collected from patients with travel history to Iraq or Syria were often associated with genotypes from Turkey, as the cgMLST profiles from these countries clustered together. Sixty strains were located within a distance of 20 core genes to related genotypes from the publicly available database, and for eighteen isolates, the closest genotype was different by more than 50 loci. Our study showed that WGS based tools are effective in tracing back the geographic origin of infection of patients with unknown travel status, provided that public sequences from the location of the source are available.

## 1. Introduction

Brucellosis is one of the most widespread bacterial zoonosis globally. Annually, half a million cases of human brucellosis are reported worldwide, but the actual number of cases is estimated to be ten times higher [1,2]. Majority of human cases are caused by *Brucella melitensis*, which is most frequently transmitted through direct contact with infected goats and sheep and through consumption of unpasteurized milk and dairy products contaminated with the bacteria [3,4,5].

Although the mortality rate of brucellosis is low, it is often debilitating and difficult to diagnose as its clinical presentation may mimic variety of infectious and noninfectious illnesses [6]. The disease can affect any organ system, and the most frequently reported symptoms include pyrexia, arthritis, and fatigue. Unspecific clinical presentation and thus the failure of correct primary diagnosis and treatment often leads to disease progression with genitourinary, neurological, pulmonary, or cardiovascular system involvement, with endocarditis being the most serious of the complications [4,6,7]. Therefore, for a prompt diagnosis, particular attention should be given to the patient’s history of travel to brucellosis endemic areas and consumption of raw milk products.

Ovine and caprine brucellosis has been eradicated in majority of the countries in European Union (EU). A few notable exceptions include Greece, Italy, Spain, and Portugal, where the annual number of confirmed human infections is the highest [8]. In 2017, ~65% of brucellosis cases reported in EU were acquired in Europe, whereas 12% were imported from other countries. Of the latter, the most common travel destinations were Iraq, Turkey, and Syria [8]. Interestingly, the country of infection or travel status was unknown for over 22% of all notified cases. Although in Europe the number of *B. melitensis* positive flocks in recent years has been gradually decreasing, in the countries of the Middle East, Central Asia, and Africa, the disease is still on the rise [1,9,10]. Therefore, international immigration trends and travel to these regions along with consumption of local contaminated foods and illegal import of dairy products pose a risk of brucellosis infection and augment the number of cases reported in EU [11,12]. Continuous surveillance programs vital for tracing the origin of human infections exist in Europe but they largely rely on effective molecular typing procedures as well as on the availability of good quality, accurate data collected nationally and internationally [13,14,15].

Both traditional and modern typing methods are used in epidemiology of Brucella. Biotyping currently recommended by World Organisation for Animal Health (OIE), divides *B. melitensis* into three biovars and relies on serological reactions of the main surface antigens with monospecific sera. The method is laborious and requires live bacteria handling and the results are often insufficient for epidemiological purposes. Therefore, molecular methods such as Multilocus Sequence Typing (MLST), Multiple Loci VNTR (Variable Number of Tandem Repeats) Analysis (MLVA), and more recently, whole genome sequencing (WGS) typing methods are additionally used to discriminate between *B. melitensis* strains, provide higher resolution genetic clustering, and identify outbreak cases [14,16,17,18].

In our work, we sequenced 103 *B. melitensis* isolates collected in Sweden in the period between 1994 and 2016. The aim of our study was to identify the putative geographic origins of the strains isolated from Swedish patients with unknown history of travel to endemic areas using three different typing methods based solely on WGS analysis.

## 2. Materials and Methods

### 2.1. Bacterial Strains and Growth Conditions

The *B. melitensis* isolates were collected between 1994 and 2016 from Swedish patients that had returned from Brucella endemic countries, stored at the biorepository of the Public Health Agency of Sweden, and used as stipulated in the regulations for diagnostic development and quality assessment. All the *B. melitensis* human clinical strains were isolated in the BSL-3 laboratory by cultivation of samples on 5% sheep blood agar plates in a 5–10% CO_2_ atmosphere at 37 °C for 48 h. The isolates used in this study were confirmed as *B. melitensis* by general real-time PCR that amplifies DNA of all Brucella strains and a specific real-time PCR for *B. melitensis*, as well as by Matrix-Assisted Laser Desorption/Ionization Time-of-Flight Mass Spectrometry (MALDI-TOF MS), as previously described [19,20,21].

### 2.2. Whole Genome Sequencing

Bacterial DNA was extracted using the commercially available EZ1^®^ DNA Tissue Kit from Qiagen, Stockholm, Sweden, according to the manufacturer’s instructions and stored at 4 °C until use. As a process control of an extraction step, 5 µL of seal herpes virus cell culture was spiked in a total volume of 200 µL of each sample. The samples were eluted in 50 µL of elution buffer.

Total genomic DNA extracted from bacterial colonies was quantified with the Qubit fluorometer (QubitTM DNA HS assay; Life Technologies, Thermo Fisher Scientific Inc., Waltham, MA, USA). Sequencing libraries were prepared using Nextera XT library preparation kit (Illumina Inc., San Diego, CA, USA) according to the manufacturer’s instructions. The libraries were sequenced using the Illumina NextSeq 500 platform, producing 150-bp paired-end reads. After demultiplexing and removal of adapters, reads were trimmed from 5′ and 3′ ends using Trimmomatic tool version 0.36 to discard the nucleotides with quality scores of less than 25. Reads shorter than 36 bp were automatically discarded. Scaffolds were assembled with SPAdes version 3.11.1 with the careful option selected [22].

Read sequences were submitted to Sequence Read Archive of the National Center for Biotechnology Information (NCBI) under the BioProject accession number PRJNA551091.

### 2.3. In Silico MLVA Typing

We extracted the MLVA-16 profiles directly from genome assemblies using MLVA In Silico Typing Resource for Salmonella Strains (MISTReSS, https://github.com/Papos92/MISTReSS#mistress-mlva-in-silico-typing-resource-for-salmonella-strains). MISTReSS tool was customized for extraction of Brucella MLVA-16 panel using previously described set of primers [23] with one modification. In order to avoid multiple primer binding sites, we extended the sequence of the forward primer of Bruce21 locus to 52 bp (5′-GGCAGTGGGGCAGTGAAGAATATGGTCGCTGCGCTCATGCGCAACCAAAACA-3′). Extracted profiles were then loaded and clustered with MLVA-8 and MLVA-16 panels in Bionumerics 7.6.3 (Applied Maths NV, Sint-Martens-Latem, Belgium) using MST (Minimum Spanning Tree) method. Additional 2535 *B. melitensis* profiles from the public and private MLVA repositories were used for identifying related genotypes.

### 2.4. MLST and cgMLST Analysis

Genome assemblies produced in our study, along with 139 public genomes available at GenBank (with known geographical origin; accessed on 30 November 2018), were genotyped using cgMLST. The cgMLST profiles were assigned using *B. melitensis* task template with 2704 target core genes in Ridom SeqSphere+ software, v4.1.1 (Ridom GmbH, Münster, Germany) as described by Janowicz and colleagues (2018) [18]. Multiple spanning tree (MST) was generated by pairwise comparison of cgMLST target genes using default parameters. Missing values were ignored in the calculation of distance between pairs of sample profiles. All sequences were additionally typed using the Brucella 9 locus Multilocus Sequence Typing (MLST-9) scheme available at https://pubmlst.org/brucella/ [24] accessible through Ridom SeqSphere+. Complete MLST-9 allele profiles of *B. melitensis* strains available in the public database and containing information about geographic origin of the strain (*n* = 179) were downloaded from https://pubmlst.org/brucella/ (accessed on 22 May 2019). MLST-9 profiles were analyzed using the goeBURST algorithm implemented in PHYLOViZ, version 2.0 [25]. Minimum spanning trees (MST) were created using default software settings.

## 3. Results

Out of 103 *B. melitensis* strains isolated from patients in Sweden between years 1994 and 2016, 71 had no available information about the country where the infection was contracted (Table 1). The majority of samples (*n* = 85) belonged to biovar 3, whereas fifteen were classified as biovar 2 and only three as biovar 1. Biotyping however, was not sufficient to determine the plausible geographic origin of the strains. Interestingly, patients who had traveled to Iraq acquired either one of the biovars.

**Figure 1:**
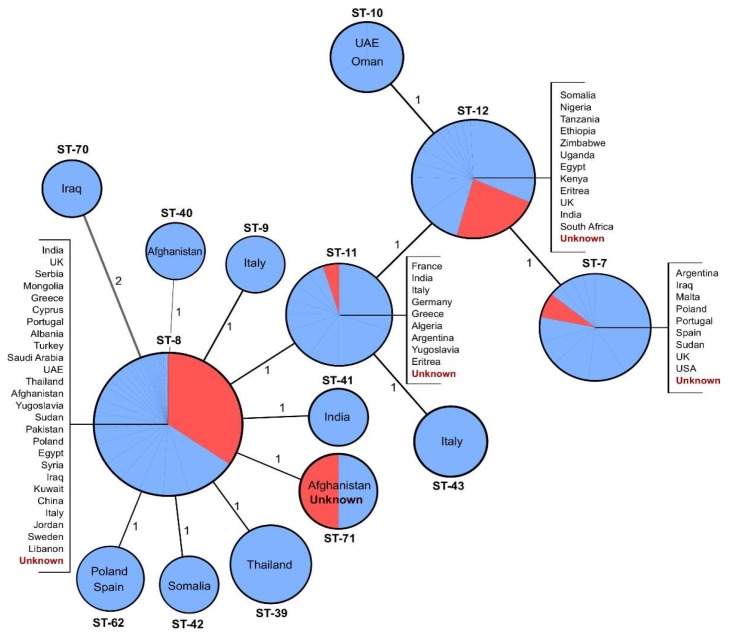
Minimum spanning tree of *B. melitensis* isolates typed by multilocus sequence typing (MLST-9). Published MLST-9 profiles (*n* = 179) downloaded from the PubMLST Database (https://pubmlst.org/brucella/) and profiles of the strains from this study (*n* = 103) were used to generate the tree using the goeBURST algorithm in PHYLOViZ software. Each node corresponds to a sequence type (ST), and the branches are labeled with the number of discriminating loci. Swedish isolates obtained from patients with unknown travel history are represented in red.

Using WGS assemblies, we were able to determine MLST profiles for all sequenced genomes. The MLST-9 typing scheme divided the Swedish *B. melitensis* isolates into five sequence types (ST-7, ST-8, ST-11, ST-12, and ST-71). We retrieved publicly available MLST-9 profiles of *B. melitensis* samples that included complete “country” information (*n* = 179). The results of MST clustering are shown in Figure 1. The largest cluster, ST-8, contained 83 of the Swedish isolates, 55 of which with unknown geographic origin. It was, however, impossible to determine the geographic links of ST-8 as it clustered strains from European, Asian, and African countries. Similarly, ST-7 and ST-11 contained isolates from more than one continent. Sequence type 12 was the second largest group and consisted of samples collected mainly in Africa, including two isolated from Swedish patients with travel history to Somalia and Ethiopia. ST-71 was present in Afghanistan.

**Figure 2:**
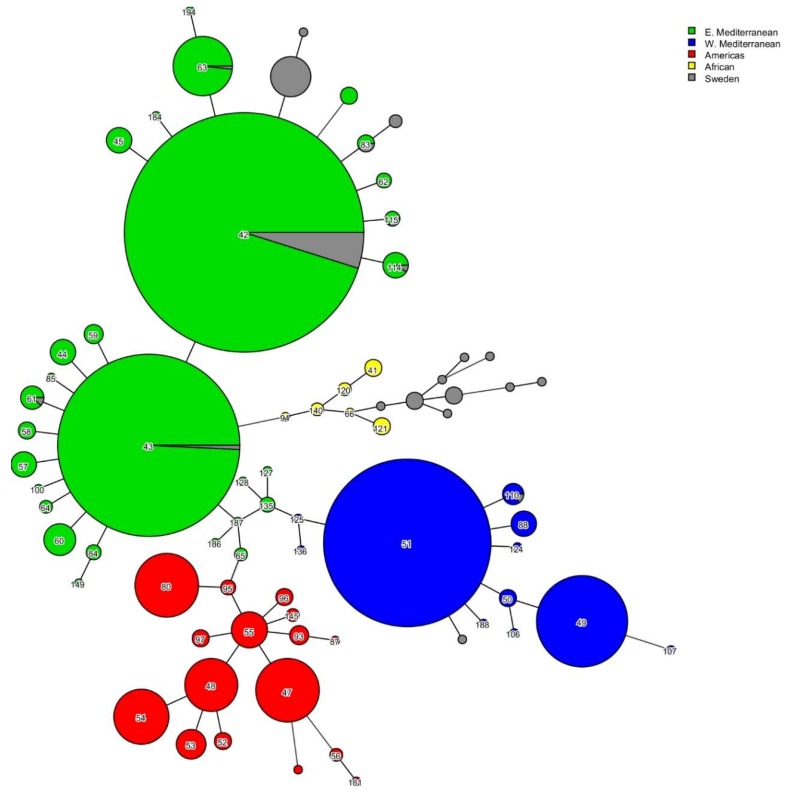
Minimum spanning tree (MST) based on *B. melitensis* MLVA-8 typing results. The tree was generated using 2535 publicly available MLVA profiles and 103 profiles of Swedish isolates calculated in silico. The nodes are labeled with the genotype and colored according to genetic lineage. MLVA-8 types obtained from strains characterized in this study are shown in gray.

The MISTReSS tool could recover the VNTRs for the 103 *B. melitensis* analyzed; however, 43 isolates were missing at least one allele calling. Bruce 43 was absent for 31 isolates and other VNTR null calls were also found for Bruce 06, Bruce 42, Bruce 19, Bruce21, Bruce 07, Bruce 09, Bruce 16, and Bruce 30. Allele 2 of Bruce 06 locus, which was present in only few publicly available *B. melitensis* MLVA profiles, was identified in sixteen strains from our dataset. Considering that MLVA-8 genotype was complete for 68 of the 103 Swedish isolates, the MST from the MLVA-8 panel was used to assign twenty different genotypes for these isolates (Figure 2). The majority of the profiles were clustered within the East Mediterranean lineage; sixteen belonged to African lineage and two were found in the West Mediterranean clade. Fifty-six isolates, all with complete MLVA-8 profiles, belonged to eight known genotypes (Figure 2), most of which (*n* = 45) were genotype 42 (GT42). Despite the incompleteness of MLVA-16 profiles, they were clustered with MST to determine the plausible geographic origin of the strains by identifying the closest genotype with known provenance (Table 1). Out of 32 isolates with known origin of infection, thirteen were linked to China according to MLVA-16 typing. Additional 26 Swedish *B. melitensis* strains of unknown provenance were also predicted to originate in China, the majority with less than three loci distance from the nearest publicly available genotype. Several strains were placed within Saudi Arabia/China/Turkey/Belgium and India/Turkmenistan/China clusters. These genetic links were not exact, as at least one VNTR of difference was observed between profiles of the Swedish isolates and the publicly available profiles. We were only able find identical MLVA-16 profiles for two strains from our study, and these were isolated in Iraq. One strain from patient that likely contracted brucellosis in Somalia was not traced back to African lineage (Table 1).

**Figure 3:**
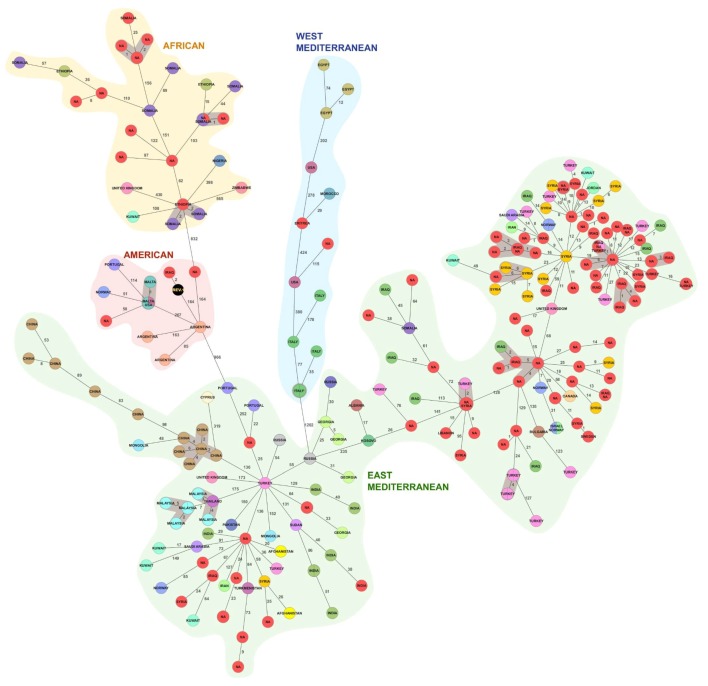
Minimum spanning tree (MST) generated for 242 isolates of *B. melitensis* using the gene-by-gene approach. MST was calculated by pairwise comparison of 2704 target genes with missing values ignored. Nodes correspond to unique profiles and are colored according to the published country of isolation and the branches correspond to the number of discriminating loci. Strains obtained from patients in Sweden are depicted in red and *B. melitensis* vaccine strain Rev.1 is shown in black. Complexes of genotypes within a distance of six alleles are highlighted in gray.

A comparison of core genome profiles of our set of samples with the publicly available genome assemblies confirmed that majority of the strains isolated in Sweden belonged to the Eastern Mediterranean clade and clustered in close proximity to *B. melitensis* genomes collected in countries of the Middle East (Figure 3). However, unlike MLVA-8 typing, cgMLST placed thirteen genotypes within African lineage and three in American. Two were clustered within West Mediterranean lineage in accordance with MLVA-8. To find the putative country of origin of the strains in our dataset, we identified the closest cgMLST genotype with known provenance (Table 1). More than a half of the isolates were located twenty or less loci away from such genomes and for sixteen strains of unknown origin we identified at least one neighbor within previously established 6-loci threshold estimated for a putative outbreak cluster (Table 1). Interestingly, we found that several genetically related strains were isolated in Turkey, Syria, and Iraq or obtained from patients with a travel history to these countries suggesting likely exchange of infected animals or contaminated milk products between these regions.

Seventeen putative outbreak clusters defined by single linkage distance of six gene variants and containing at least three isolates were identified in the cgMLST dataset, out of which, eleven contained at least one strain isolated in Sweden. We found not only that closely related strains could originate from different countries, but additionally some of them persisted in animal populations for long time periods. For instance, in Complex 2, sample 2017-TE-24378-1-12 was collected in 1998 and four other strains were isolated in 2011 and 2015. On the contrary, some complexes, e.g., Complex 12, included several strains genetically related to a Somalian isolate, which were all collected in the same year and possibly originated in the same location.

The isolate 2017-TE-24378-1-90, from a patient with a history of travel to Iraq, was found within American clade. We compared the cgMLST profile of this isolate with a Rev.1 vaccine strain (GCA_002953595.1), which also belongs to the American lineage, and found only two loci of difference between the two profiles confirming that the infection was caused by the vaccine strain (Figure 3).

## 4. Discussion

In Europe, brucellosis has often been considered a zoonosis affecting mainly veterinarians and livestock owners and breeders. However, increased tourism, migration, and emerging trends towards the consumption of local and raw products have led to changes in epidemiology of human brucellosis and raised the necessity for enhanced surveillance and control programs worldwide.

Most European Union member states, including Sweden, are currently classified as officially free from *B. melitensis* infection and therefore human brucellosis is considered an imported disease [8]. From epidemiological point of view, it is therefore necessary to trace back the origin of the infection, particularly in cases where history of travel to Brucella endemic regions is unknown.

Our study used WGS based tools combined with associated publicly available databases to determine possible geographic origins of *B. melitensis* isolates cultured from patients in Sweden. We found that majority of strains belonged to East Mediterranean and African clades. Only five cases were attributed to West Mediterranean and American lineages. The isolates most frequently originated in Middle East and were genetically related to strains from Iraq, Syria, and Turkey in particular. Within the African clade, we found that Somalia was the most likely place where the patients contracted brucellosis. This observation is supported by the demographic composition in Sweden, where Iraqi, Syrians, and Somalians are among the ten largest groups of foreign-born persons, and both new immigrants and travelers visiting their families in these territories likely augment the numbers of cases of brucellosis in Sweden. Other studies have reported similar results, demonstrating that human *B. melitensis* infection diagnosed in Europe is most frequently acquired in countries of the Middle East, where ovine and caprine brucellosis is endemic [26,27,28,29,30]. The majority of our samples were assigned to the biovar 3, which has been reported to be a predominant *B. melitensis* biovar isolated from animals and humans in Middle East [27,29,30]. We additionally observed that two closely related isolates, one of which originating in Turkey, belonged to two different biovars. Traditional typing methods are laborious, require skilled technical staff, and good quality reagents and are more prone to erroneous or inconclusive results than molecular typing techniques. The procedure can therefore inadvertently lead to the assignment of a wrong biovar. Moreover, it has been demonstrated that traditional biotyping results may not strictly reflect genetic nor phylogeographic clustering of *B. melitensis* [31]. Thus, higher resolution molecular typing techniques are now preferentially used for epidemiological studies of Brucella.

Several typing methods can be utilized in molecular epidemiology of brucellosis and our study compared the effectiveness of MLST, MLVA in silico and cgMLST in identifying putative geographic source of *B. melitensis* strains based on the genetic profile similarities. We found that MLST had little discriminatory power; however, it was still superior to the standard biotyping technique, particularly for identification of the strains likely originating in Africa. MLST has been previously used to describe genetic diversity of Brucella in Asia [32,33], however this method might be more appropriate for deeper phylogenetic points, such as distinction between the species rather than provenance of specific genotypes [34].

The MISTReSS tool could not recover all Variable Number of Tandem Repeats (VNTRs) creating possible genotyping errors especially considering the clusters generated with the smaller MLVA-8 panel that included the set of more stable genetic markers. The probable clustering errors were reduced by using the wider MLVA-16 panel. For majority of the isolates, MLVA-16 assigned the putative origin of the Swedish *B. melitensis* strains to China or to East Mediterranean clusters that included strains isolated in China and several other countries. Links to Chinese *B. melitensis* isolates were also observed for strains obtained from patients with known travel history to Middle East. This suggests that MLVA might not have enough resolution to discern between some strains of East Mediterranean lineage or that the public collection of MLVA profiles is insufficient to find some of the truly related strains. Indeed, we were only able to identify two exact MLVA-16 profile matches to our isolates in the public database.

Gene-by-gene analysis of genome assemblies and comparison of the cgMLST profiles allowed identification of at least one neighboring isolate with known geographic origin located within a distance of six alleles for sixteen of the strains from Swedish patients. This would suggest a high probability that these strains originated from the same region. Interestingly, we found that very closely related strains were often isolated from travelers returning from Turkey, Syria, and Iraq. As the three countries share their borders, people may travel through more than one of the brucellosis endemic regions to reach their destination. Moreover, it is likely that the infected animals or contaminated products are often exchanged between the bordering territories. Indeed, in recent years, due to human migration from Syria, an estimated 100,000 of sheep and goats were introduced to Northern provinces of Iraq [35]. Illegal animal movements undermine national brucellosis control programs and are a serious risk for public health, particularly where unpasteurized dairy products are frequently consumed.

Using WGS analysis, we were able to identify a likely case of infection with *B. melitensis* Rev-1 vaccine strain that had been previously demonstrated to be pathogenic in human [36]. Sheep vaccination against *B. melitensis* is recommended in Iraq and several mass vaccination campaigns were undertaken in the past [37]. The patient with the history of travel to Iraq must have, therefore, acquired the infection from the vaccinated animals or their products. The live attenuated Rev.1 strain is widely used to control brucellosis in small ruminants and, when properly administered, it provides long lasting protection against disease. However, in pregnant animals, the bacteria can still be shed in milk and thus consuming unpasteurized milk and dairy products from recently immunized animals poses a high risk of infection [36]. Additionally, WGS analysis suggested a link between two isolates which shared the same cgMLST profile, one from a returning traveler to Iraq and a person with no travel history to brucellosis endemic areas. Further inquiry revealed that the infection acquired in Sweden was a healthcare worker who got infected while processing the sample from the other patient [38].

Molecular typing methods are essential in control and surveillance of infectious diseases. Here, we showed the utility of WGS-based tools, but currently their use requires specialized personnel and equipment for genome sequencing and bioinformatic analyses that may be limited to larger institutes and to reference centers. Traditional MLVA, available in many of laboratories, might therefore still be used as a first line assay, as it offers sufficient discriminatory power for the routine brucellosis surveillance programs [14,16,39].

Despite the superior resolution of cgMLST compared to the other tools used in our study, we did not find closely related strains to at least eighteen isolates which differed from the other strains by at least 50 target genes. This highlights the limitations imposed by the lack of publicly available sequences from *B. melitensis* endemic regions. In fact, a large proportion of deposited data are obtained from human infections imported from abroad and in many cases the epidemiological data, such as host, year and location of pathogen isolation are missing. Moreover, for human isolates where the strain isolation country is known, it might not coincide with the location where the infection was contracted. Additionally, the sequences of *B. melitensis* strains isolated from small ruminants are lacking, partially due to lack of resources for application of WGS technology in developing countries and, secondly, due to a lesser impact of animal infection epidemiology compared to human brucellosis research. In order to facilitate programs for effective control and surveillance of brucellosis worldwide an international effort should be made to provide sufficient data for effective tracing of *B. melitensis* in the world and in particular in the regions where ovine and caprine brucellosis is currently endemic or re-emerging.

## Figures and Tables

**Table 1:** *B. melitensis* isolates analyzed in this study.

Sample ID	BV	ST	Year	Country	Putative Origin (MLVA-16)	MLVA-16 Distance	VNTRs Recovered MLVA-16	Putative Origin (WGS)	Core Genome Distance	cgMLST Complex
2017-TE-24378-1-1	3	11	1994	NA	France	2	16	USA	115	
2017-TE-24378-1-2	3	8	1994	NA	China	2	16	Somalia	64	
2017-TE-24378-1-3	3	12	1995	NA	Turkey/China	6	16	Somalia	127	
2017-TE-24378-1-4	2	8	1995	NA	Iraq	0	16	Afghanistan	20	
2017-TE-24378-1-5	1	7	1995	NA	Peru	4	15	Argentina	164	
2017-TE-24378-1-6	3	12	1996	NA	Turkey/China	4	16	Somalia	157	C6
2017-TE-24378-1-7	3	12	1996	NA	Turkey/China	4	16	Somalia	156	C6
2017-TE-24378-1-8	3	12	1996	NA	Turkey/China	4	16	Somalia	157	C6
2017-TE-24378-1-9	3	12	1996	NA	Turkey/China	4	16	Somalia	156	C6
2017-TE-24378-1-10	3	8	1997	NA	Saudi Arabia/China/ Turkey/Belgium	3	15	Syria	9	
2017-TE-24378-1-11	1	7	1998	NA	Germany	4	14	Malta	58	
2017-TE-24378-1-12	3	8	1998	NA	China	3	15	Iraq	5	C2
2017-TE-24378-1-13	3	8	2000	NA	China	1	16	Kosovo	26	
2017-TE-24378-1-14	3	8	2001	NA	China	2	16	Syria	9	
2017-TE-24378-1-15	2	8	2002	NA	Spain	1	16	Portugal	22	
2017-TE-24378-1-17	3	8	2002	NA	China	1	16	Syria	12	
2017-TE-24378-1-19	3	8	2002	NA	Saudi Arabia/China/ Turkey/Belgium	1	16	Syria	8	
2017-TE-24378-1-20	3	8	2003	NA	Iraq	0	16	Syria	6	C9
2017-TE-24378-1-21	3	8	2003	NA	Saudi Arabia/China/ Turkey/Belgium	1	16	Syria	9	
2017-TE-24378-1-22	3	8	2004	NA	China	1	16	Syria	11	
2017-TE-24378-1-23	3	8	2004	Syria	South Africa	4	15	Turkey	97	
2017-TE-24378-1-24	3	8	2004	Iraq	China	2	15	Turkey	8	
2017-TE-24378-1-25	3	12	2005	Ethiopia	Somalia	5	13	Somalia	2	C7
2017-TE-24378-1-26	3	8	2005	NA	China	2	15	Syria/Iraq	16	C3
2017-TE-24378-1-27	3	8	2005	NA	India/ Turkmenistan/ China	3	15	Turkey	14	C15
2017-TE-24378-1-28	3	8	2005	NA	India/China	1	16	Turkey	10	C8
2017-TE-24378-1-29	3	12	2005	Somalia	Turkey/China	7	16	Somalia	161	
2017-TE-24378-1-30	3	8	2005	NA	Kazakhstan/China	1	16	Iraq	28	
2017-TE-24378-1-31	3	8	2005	Iraq	China	1	16	Syria	11	
2017-TE-24378-1-32	3	12	2008	NA	Spain	5	15	Ethiopia	36	
2017-TE-24378-1-33	3	8	2006	NA	China	3	15	Iraq	21	
2017-TE-24378-1-34	3	8	2006	NA	China	1	16	Turkey	9	
2017-TE-24378-1-35	3	8	2006	Syria	India/ Turkmenistan/ China	2	16	Syria/ Turkey	23	
2017-TE-24378-1-36	3	8	2006	NA	India	1	16	Turkey	8	
2017-TE-24378-1-37	2	8	2007	NA	Saudi Arabia/China/ Turkey/Belgium	3	16	India	73	
2017-TE-24378-1-38	3	8	2007	NA	Saudi Arabia/China/ Turkey/Belgium	1	16	Syria	11	C17
2017-TE-24378-1-39	3	8	2007	Syria	Saudi Arabia/China/ Turkey/Belgium	1	16	Syria	11	C17
2017-TE-24378-1-40	3	8	2007	NA	Saudi Arabia/China/ Turkey/Belgium	1	16	Syria	11	C17
2017-TE-24378-1-41	2	8	2007	Iraq	China	1	16	Syria	24	
2017-TE-24378-1-42	3	8	2007	Syria	China	2	15	Turkey	11	
2017-TE-24378-1-43	3	8	2008	NA	India/ Turkmenistan/ China	2	16	Turkey	14	C15
2017-TE-24378-1-45	3	8	2008	NA	China	2	16	Iraq	16	
2017-TE-24378-1-46	3	8	2008	NA	China	2	16	Iraq	32	
2017-TE-24378-1-47	3	8	2009	Iraq	China	1	16	Syria/Iraq	9	
2017-TE-24378-1-48	3	12	2009	NA	Spain	9	16	Somalia	101	
2017-TE-24378-1-49	3	8	2009	NA	China	1	16	Turkey	12	
2017-TE-24378-1-50	3	8	2009	Iraq	Saudi Arabia/China/ Turkey/Belgium	1	16	Syria/Iraq	17	C3
2017-TE-24378-1-51	3	8	2010	NA	India/ Turkmenistan/ China	3	14	Turkey	7	C4
2017-TE-24378-1-52	3	8	2010	NA	China	4	15	Somalia	38	
2017-TE-24378-1-53	3	8	2011	NA	Saudi Arabia/China/ Turkey/Belgium	1	16	Syria/Iraq	20	C3
2017-TE-24378-1-54	3	8	2011	Syria	China	3	16	Iraq	23	
2017-TE-24378-1-55	3	8	2011	Sweden	China	3	16	Iraq	24	
2017-TE-24378-1-56	2	8	2011	India	India/China	1	16	India	38	
2017-TE-24378-1-57	3	8	2011	NA	China	2	15	Iraq	5	C2
2017-TE-24378-1-58	3	8	2011	Iraq	Saudi Arabia/China/ Turkey/Belgium	2	15	Syria, Iraq	17	C3
2017-TE-24378-1-59	3	8	2011	NA	Saudi Arabia/China/ Turkey/Belgium	1	16	Syria/Iraq	17	C3
2017-TE-24378-1-60	3	8	2011	NA	China	2	15	Iraq	21	
2017-TE-24378-1-62	2	8	2011	NA	China	3	16	Georgia	33	
2017-TE-24378-1-63	3	8	2012	NA	Kazakhstan/ China	1	16	Syria	14	
2017-TE-24378-1-64	3	8	2012	NA	China	2	15	Iraq	7	
2017-TE-24378-1-65	3	8	2012	Iraq	China	1	15	Turkey	13	
2017-TE-24378-1-66	3	8	2012	NA	Kazakhstan/China	2	15	Iraq	29	
2017-TE-24378-1-67	3	8	2012	NA	China	2	14	Turkey	13	
2017-TE-24378-1-68	3	8	2012	NA	Kazakhstan/ China/Mongolia	3	15	Afghanistan	32	
2017-TE-24378-1-69	3	12	2012	NA	Spain	8	15	Somalia	1	C12
2017-TE-24378-1-70	2	8	2012	Turkey	China	2	15	Syria/Turkey	24	
2017-TE-24378-1-71	3	8	2012	Lebanon	Kazakhstan/ China	3	15	Turkey	15	
2017-TE-24378-1-72	3	8	2012	Iraq	India/ Turkmenistan/ China	1	16	Syria	20	
2017-TE-24378-1-73	3	8	2013	NA	India/ Turkmenistan/ China	2	15	Syria	20	
2017-TE-24378-1-74	3	8	2013	Iraq	India/ Turkmenistan/ China	3	16	Turkey	15	C15
2017-TE-24378-1-75	3	8	2013	NA	Saudi Arabia/China/ Turkey/Belgium	1	16	Syria/Iraq	19	C3
2017-TE-24378-1-76	2	8	2013	NA	Turkey	4	15	Afghanistan	37	
2017-TE-24378-1-77	3	8	2013	Turkey	China	3	15	Syria/ Turkey	28	
2017-TE-24378-1-78	2	8	2013	NA	China	2	16	Syria/ Turkey	28	
2017-TE-24378-1-79	3	12	2014	NA	Spain	8	14	Somalia	0	
2017-TE-24378-1-80	3	12	2014	NA	Spain	7	14	Somalia	64	
2017-TE-24378-1-81	2	71	2014	NA	Turkey/Iran/Iraq/ Jordan	2	15	Turkmenistan	78	
2017-TE-24378-1-82	2	8	2014	NA	Turkey	3	16	Afghanistan	37	
2017-TE-24378-1-83	3	8	2014	NA	China	3	16	Turkey	1	C4
2017-TE-24378-1-84	3	8	2014	NA	India/ Turkmenistan/ China	1	16	United Kingdom	17	
2017-TE-24378-1-85	3	8	2014	NA	India/ Turkmenistan/ China	4	15	Turkey	2	C11
2017-TE-24378-1-86	3	8	2014	Syria	India/ Turkmenistan/ China	4	15	Turkey	2	C11
2017-TE-24378-1-87	3	8	2014	Syria	India/ Turkmenistan/ China	2	15	Turkey	2	C11
2017-TE-24378-1-88	3	8	2014	NA	India/ Turkmenistan/ China	3	15	Turkey	2	C11
2017-TE-24378-1-89	3	8	2014	NA	China	3	15	Turkey	0	C4
2017-TE-24378-1-90	1	7	2014	Iraq	Israel/Portugal/ France/Italy	3	16	Argentina	164	
2017-TE-24378-1-91	3	8	2014	Iraq	China	2	16	Turkey	1	C4
2017-TE-24378-1-92	3	8	2015	Iraq	China	3	15	Turkey	9	C8
2017-TE-24378-1-93	3	8	2015	Iraq	China	2	16	Turkey	8	C8
2017-TE-24378-1-94	3	8	2015	Iraq	Saudi Arabia/China/ Turkey/Belgium	3	15	Iraq	2	C2
2017-TE-24378-1-95	3	11	2015	Eritrea	Tunisia/France	3	15	Morocco	29	
2017-TE-24378-1-96	3	8	2015	Iraq	China/India	2	15	Turkey	12	
2017-TE-24378-1-97	3	8	2015	NA	China	2	16	Syria	13	
2017-TE-24378-1-98	2	8	2015	Syria	Saudi Arabia/China/ Turkey/Belgium	1	16	Turkey/ Kuwait	76	
2017-TE-24378-1-99	3	8	2015	NA	China	1	16	Syria	14	
2017-TE-24378-1-100	3	8	2015	Syria	China/India	1	16	Turkey	4	
2017-TE-24378-1-101	3	8	2015	NA	China/India	1	16	Iraq	3	C2
2017-TE-24378-1-102	3	8	2016	NA	Saudi Arabia/China/ Turkey/Belgium	4	15	Turkey	10	
2017-TE-24378-1-103	3	12	2016	NA	Spain	4	16	Ethiopia	36	
2017-TE-24378-1-104	3	8	2001	NA	China	2	15	Turkey	9	
2017-TE-24378-1-105	2	71	2010	NA	Iraq	1	15	Turkmenistan	73	
2017-TE-24378-1-108	2	8	2011	NA	China	1	16	Syria	25	
2017-TE-24378-1-109	2	8	2011	NA	China	3	16	Georgia	33	

BV: Biovar; NA: Not available, ST: Sequence Type.
